# A randomized cross-over trial to define neurophysiological correlates of AV-101 *N*-methyl-d-aspartate receptor blockade in healthy veterans

**DOI:** 10.1038/s41386-020-00917-z

**Published:** 2020-12-14

**Authors:** Nicholas Murphy, Nithya Ramakrishnan, Bylinda Vo-Le, Brittany Vo-Le, Mark A. Smith, Tabish Iqbal, Alan C. Swann, Sanjay J. Mathew, Marijn Lijffijt

**Affiliations:** 1 Michael E. DeBakey VA Medical Center, 2002 Holcomb Boulevard, Houston, TX 77030 USA; 2 Menninger Department of Psychiatry and Behavioral Sciences, Baylor College of Medicine, 1977 Butler Boulevard, Houston, TX 77030 USA; 3 VistaGen Therapeutics, Inc., 343 Allerton Avenue, South San Francisco, CA 94080 USA; 4 Medical College of Georgia, 1120 15th Street, Augusta, GA 30912 USA

**Keywords:** Biomarkers, Neurophysiology, Neuroscience

## Abstract

The kynurenine pathway (KP) is a strategic metabolic system that combines regulation of neuronal excitability via glutamate receptor function and neuroinflammation via other KP metabolites. This pathway has great promise in treatment of depression and suicidality. The KP modulator AV-101 (4-chlorokynurenine, 4-Cl-KYN), an oral prodrug of 7-chlorokynurenic acid (7-Cl-KYNA), an *N*-methyl-d-aspartate receptor (NMDAR) glycine site antagonist, and of 4-chloro-3-hydroxyanthranilic acid (4-Cl-3-HAA), a suppressor of NMDAR agonist quinolinic acid (QUIN), is a promising potential antidepressant that targets glutamate functioning via the KP. However, a recent placebo-controlled clinical trial of AV-101 in depression found negative results. This raises the question of whether AV-101 can penetrate the brain and engage the NMDAR and KP effectively. To address this problem, ten healthy US military veterans (mean age = 32.6 years ± 6.11; 1 female) completed a phase-1 randomized, double-blind, placebo-controlled, crossover study to examine dose-related effects of AV-101 (720 and 1440 mg) on NMDAR engagement measured by γ-frequency band auditory steady-state response (40 Hz ASSR) and resting EEG. Linear mixed models revealed that 1440 mg AV-101, but not 720 mg, increased 40 Hz ASSR and 40 Hz ASSR γ-inter-trial phase coherence relative to placebo. AV-101 also increased 4-Cl-KYN, 7-Cl-KYNA, 4-Cl-3-HAA, 3-HAA, and KYNA in a dose-dependent manner, without affecting KYN and QUIN. AV-101 was safe and well tolerated. These results corroborate brain target engagement of 1440 mg AV-101 in humans, consistent with blockade of interneuronal NMDAR blockade. Future studies should test higher doses of AV-101 in depression. Suicidal behavior, which has been associated with high QUIN and low KYNA, is also a potential target for AV-101.

## Introduction

The kynurenine pathway (KP) links excitatory amino acid transmission and neuroinflammation, and is a promising target for treatment of depression and suicidality. The KP modulator AV-101 (4-chlorokynurenine, 4-Cl-KYN) is an oral prodrug of 7-chlorokynurenic acid (7-Cl-KYNA), which is converted by kynurenine aminotransferase (KAT)-rich astrocytes [1, 2] and which acts as a high-affinity *N*-methyl-d-aspartate receptor (NMDAR) strychnine-insensitive glycine-binding site competitive antagonist [3–5] that strongly inhibits NMDAR activation [5]. AV-101 is also a prodrug of 4-chloro-3-hydroxyanthranillic acid (4-Cl-3-HAA) [6], which is converted by kynurenine 3-monooxygenase-rich microglia and which acts as an inhibitor of NMDAR agonist quinolinic acid (QUIN) [7]. AV-101 is in early phase development for major depressive disorder (MDD) based on potential ketamine-like antidepressant effects and reduced potential for behavioral side effects [8]. However, a recently completed randomized, placebo-controlled, double-blind, crossover study in treatment-resistant depression (TRD) found no difference on the Hamilton Depression Rating Scale between 14 days of placebo and 14 days of AV-101 (1080 mg/day for 7 days followed by 1440 mg/day for 7 days) [9]. In that study, the first administration of AV-101 1080 mg did not change the concentration of cerebrospinal fluid (CSF) 7-Cl-KYNA, although it did increase CSF AV-101 and plasma 7-Cl-KYNA. These findings raise the question of whether oral doses of AV-101 enter the brain in adequate concentrations to demonstrate the effects of brain penetration. More specific confirmation of the mechanism of action for AV-101 requires a functional indicator of NMDAR engagement.

Electroencephalography (EEG) can provide a marker of the functional role of these receptors in behavior and of their pharmacological engagement. NMDAR inhibition is associated with increased oscillatory γ-power during intracortical electrophysiological recordings [10, 11]. These EEG γ-oscillations arise from synchronous firing of GABAergic inhibitory interneurons and are related to inhibitory interneurons [12]. Non-competitive NMDAR antagonists suppress firing of interneurons, while stimulating pyramidal neurons, consistent with blockade of interneuronal NMDARs [13]. This enhances resting [14] and auditory steady-state response (ASSR)-generated γ-band oscillations [15, 16]. In clinical studies, 40 Hz ASSR γ-power indicates dysfunctional GABAergic and NMDAR activity [17, 18], providing a potential indirect indicator of neuronal effects of AV-101. γ-Oscillations are a core component of cognitive organization that are affected across the spectrum of MDD [19, 20]. In clinical neurophysiology, the ASSR is a well-studied and robust marker of the integrity of synchronized inhibitory interneuron communication (see refs. [16, 21]). Preclinical studies demonstrate that evoked γ-activity during 40 Hz ASSR is increased at acute doses of the NMDAR antagonist ketamine [15, 22–25], highlighting that inhibition of these channels increases the synchrony of γ-oscillations, and by extension cognitive organization [22]. A neurophysiological marker will help to determine whether the lack of clinical effects recently reported [9] might be due to insufficient NMDA inhibition because of subtherapeutic dosing of AV-101 and will be valuable in assessing the roles of NMDA receptors in behavior and treatment.

In this phase-1 randomized, double-blind, placebo-controlled, crossover study in healthy military veterans, we examined dose-related effects of AV-101 (720 and 1440 mg) on KP regulation, AV-101 metabolites, and NMDAR target engagement measured by γ-band EEG, assessed by the 40 Hz ASSR and during rest. We hypothesized a dose-related relationship between drug, KP modulation, and γ-band activity.

## Materials and methods

### Ethical statement

This study is registered at ClinicalTrials.gov (NCT03583554). Study procedures were approved by the Baylor College of Medicine Institutional Review Board and the Research and Development Committee of the Michael E. DeBakey VA Medical Center (MEDVAMC) in Houston, Texas. Subjects provided written informed consent before any study-related activities were performed.

### Participants

Eighteen Operation Enduring Freedom, Operation Iraqi Freedom, Operation New Dawn, or Operation Freedom’s Sentinel veterans were recruited by advertisement and community outreach. Twelve subjects met eligibility criteria; ten subjects (mean age = 32.6 years ± 6.11; 1 female) completed study procedures (see the CONSORT chart in Supplemental Material). Participants did not meet criteria for a current Diagnostic and Statistical Manual of Mental Disorders 5 (DSM-5) psychiatric disorder as per the Mini International Neuropsychiatric Interview [26], had a normal EKG and metabolic panel, and had no current substance (including nicotine) or alcohol-use disorder. No subjects had a serious or unstable medical condition or history of traumatic brain injury. Subjects had negative urine drug toxicologies and pregnancy screens at the time of study procedures. Full details of the inclusion and exclusion criteria, as well as losses and exclusions from the study, are reported in the Supplementary Material.

### Study design

We conducted a randomized, double-blind, placebo-controlled crossover trial to examine effects of a single dose of AV-101 (720 or 1440 mg) and placebo on γ-band oscillations and KP metabolites between 9 November 2018 and 22 October 2019. The placebo used was a weight-matched orange Capsugel Coni-Snap Size 00 capsules containing microcrystalline cellulose (Avicel PH-101). Dose selection was based on outcomes from a Phase-1 dose-escalation study in healthy volunteers [27]. Maximum plasma concentrations of AV-101 and 7-Cl-KYNA across the tested doses (360, 720, 1080, and 1440 mg) were reached 1 to 2 h after intake; half-life was between 1.5 and 2 h [27]. Plasma concentration of AV-101 and 7-Cl-KYNA clearly separated between 1440 and 720 mg AV-101, but not between 1440 and 1080 mg. All doses were well tolerated [27].

Randomization was performed by a research pharmacist, who had no patient contact, by randomly permuting the three dose conditions for each subject to generate the dose for the first week. The second and third week doses were then assigned according to the first week dose, such that week 1 = 1440 mg was followed by Placebo for week 2 and 720 mg for week 3; week 1 = 720 mg was followed by 1440 mg for week 2 and placebo for week 3; and Week 1= placebo was followed by 720 mg for week 2 and 1440 mg for week 3. Only the pharmacist had access to the randomization code; clinicians, raters, and data analysts were masked to the treatment group. For this study, drugs were given on three separate test days with at least 5 days (median interval: 13 days; range: 5–77 days) between doses. Study procedures started at about 8:00 a.m. after an overnight fast. At each visit, subjects took four indistinguishable oral capsules, each containing 360 mg AV-101 or placebo provided to the research pharmacy by VistaGen Therapeutics. EEG, blood samples for pharmacokinetic analyses, and the Profile of Mood States (POMS) [28] were collected before (pretreatment baseline) and at hourly intervals for 5 h after drug intake. Blood pressure and pulse were collected before and at 15 min intervals for 5 h after drug intake. Adverse events were assessed at hourly intervals for 5 h and at ~24 h after drug intake.

### Outcome measures

During testing, subjects rested on a bed in a 50–70° supine position. EEG was recorded using Curry 7.0.10 software by a SynAmps-RT 64-channel amplifier (Compumedics Neuroscan, Charlotte, NC, USA) and a 64-channel actiCAP (Brain Vision, Morrisville, NC, USA) with maximum channel impedance of 10 kΩ. Resting EEG activity was collected by two series of alternating 1 min eyes closed and 1 min eyes open. The ASSR was collected in three blocks that each consisted of 110 trials of 500 ms click trains composed of 1 ms-duration clicks (1000 Hz, 80 dB) repeated at frequencies of 40, 30, or 20 Hz [16, 17]. Inter-trial interval was 3 s. Click trains were presented binaurally through ER-3A insert earphones (Etymotic Research, IL, USA). To monitor participant engagement/attention, subjects counted randomly presented oddball click trains (eleven 2000 Hz tones); oddball trials were not used in the final data analysis. EEG data were processed using in-house Matlab scripts and routines adapted from the EEGLab toolbox [29]. EEG pre-processing and feature extraction are described in full in the Supplemental Material. The Matlab code to process and extract features from the data is available at (https://github.com/NikMNclUth/AV101-EEG).

KP metabolites 4-Cl-KYN, 7-Cl-KYNA, 4-Cl-3-HAA, l-kynurenine (KYN), kynurenic acid (KYNA), 3-hydroxyanthranillic acid (3-HAA), and QUIN were analyzed in blood plasma by Quintara Discovery, Inc. (Hayward, CA). Blood samples (4 ml) for analyses of metabolites were collected with a catheter placed in a vein of the hand or arm. Samples were centrifuged directly after collection for 10 min at 3100 r.p.m. Plasma was separated from serum and stored at −70 °C until assayed at the end of the study. Full details on assays and pharmacokinetics are provided in Supplemental Material (see also Tables S1–S3).

### Statistics

Dose-related differences in measures were estimated using linear mixed models (LMMs). Analyses of EEG measures used data collected from 1 to 4 h post administration because of high levels of noise in the data for the 5 h post-administration timepoint. Our model contained drug dose (placebo, low dose, and high dose) as a fixed effect and a random intercept per subject. Time (1 to 4 h post administration) and pretreatment baseline measurement were included as covariates. For KP metabolites, vital signs, and POMS, LMM analyses were performed across 1–5 h post administration with pretreatment baseline measurement as covariate. Statistical analyses were conducted using SPSS (version 26, Armonk, NY: IBM Corp). Data collection and analyses were conducted blind to treatment condition.

## Results

Recruitment baseline demographics are reported in Table 1. Outcomes of mixed model analyses are displayed in Table 2.

**Table 1:** Summary of the baseline demographics for participants recruited to the study.

ID	Age	Gender	Race	BMI	Completed study
AV101-04	26	Male	White/H	30	No
AV101-05	25	Male	White/H	25.6	Yes
AV101-06	39	Female	AA/NH	23.6	Yes
AV101-07	24	Male	White/H	31.1	No
AV101-08	37	Male	White/H	26.3	Yes
AV101-09	29	Male	White/H	31	Yes
AV101-10	29	Male	White/NH	21.5	Yes
AV101-13	37	Male	White/H	27.7	Yes
AV101-14	33	Male	White/NH	26.6	Yes
AV101-15	28	Male	White/NH	38.2	Yes
AV101-17	26	Male	White/H	30.2	Yes
AV101-18	43	Male	AA/NH	24.6	Yes
Average	31.33 ± 6.28			28.03 ± 4.42	

In the column “Race,” H/NH refers to Hispanic/Not Hispanic, respectively, AA is used to denote African American.

**Table 2:** An overview of the linear mixed model results for the neurophysiology, metabolite, vital sign, and the profile of mood states (POMS) data.

Variable		Mean (±SE)			Fixed effect		Low vs. placebo				High vs. placebo			
		Placebo	Low dose	High dose	F	*P*	*T*	*P*	Lower 95% CI	Upper 95% CI	*T*	*P*	Lower 95% CI	Upper 95% CI
40 Hz ASSR	Power*	0.34 (±0.11)	0.43 (±0.11)	0.59 (±1.1)	5.26	0.01	1.23	0.22	−0.17	0.73	3.21	<0.001	0.28	1.18
	ITPC*	0.18 (±0.02)	0.22 (±0.02)	0.2 (±0.02)	3.10	0.05	1.82	0.07	0.00	0.10	2.38	0.02	0.01	0.12
30 Hz ASSR	Power	0.46 (±0.08)	0.64 (±0.08)	0.42 (±0.08)	0.09	0.91	0.42	0.67	−0.36	0.56	0.30	0.77	−0.39	0.53
	ITPC	0.104 (±0.002)	0.105 (±0.002)	0.108 (±0.002)	4.34	0.02	−0.77	0.44	−0.02	0.01	2.07	0.04	0.00	0.03
20 Hz ASSR	Power*	0.62 (±0.07)	0.42 (±0.07)	0.61 (±0.07)	3.07	0.05	−1.87	0.06	−0.58	0.02	0.45	0.65	−0.23	0.37
	ITPC	0.14 (±0.005)	0.15 (±0.005)	0.14 (±0.005)	2.02	0.14	0.10	0.92	−0.02	0.02	1.79	0.08	0.00	0.04
Resting	Power*	−50.61 (±0.48)	−50.89 (±0.47)	−50.45 (±0.47)	5.14	0.01	1.18	0.24	−1.21	4.77	−1.98	0.05	−5.98	0.01
	Slope	−0.97 (±0.06)	−1.04 (±0.07)	−0.92 (±0.07)	1.78	0.17	−1.76	0.08	−0.55	0.03	−0.28	0.78	−0.33	0.25
KP metabolites	3-HAA*	3.35 (±0.73)	4.33 (±0.74)	6.38 (±0.74)	8.63	<0.001	1.47	0.15	−0.66	4.46	4.10	<0.001	2.75	7.86
	Kynurenic acid*	6.05 (±1.54)	8.8 (±1.55)	10.5 (±1.55)	3.56	0.03	1.86	0.06	−0.23	7.94	2.58	0.01	1.24	9.38
	Kynurenine	296.93 (±27.61)	335.1 (±27.76)	322.08 (±27.72)	2.30	0.10	1.88	0.06	−3.45	130.00	0.03	0.98	−65.77	67.54
	Quinolinic acid	47.05 (±3.39)	50.25 (±3.36)	51.84 (±3.35)	2.92	0.06	2.35	0.02	1.87	21.74	0.69	0.49	−6.48	13.35
AV-101 metabolites	4-CL-3-HAA*	3.8 (±2.95)	12.8 (±3.03)	19.59 (±3.04)	6.14	0.00	1.54	0.13	−3.52	27.92	3.50	0.00	12.13	43.68
	7-CL-KYNA*	2.4 (±27.83)	90.88 (±28.41)	168.38 (±26.47)	3.33	0.04	1.70	0.09	−19.33	258.00	2.53	0.01	38.56	315.71
	4-CL-KYN*	228.94 (±1988.1)	18092.86 (±2029.32)	26628.56 (±2064.37)	20.34	<0.001	5.60	<0.001	21925.04	45880.60	5.42	<0.001	20966.67	45070.22
POMS	Total	11.59 (±1.65)	12.03 (±1.65)	11.58 (±1.65)	1.74	0.18	−1.80	0.07	−5.34	0.24	−1.31	0.19	−4.65	0.93
	Elation total	3.61 (±0.58)	3.77 (±0.58)	3.63 (±0.58)	2.35	0.10	−2.17	0.03	−3.69	−0.17	−1.15	0.25	−2.78	0.73
Vitals	Pulse*	68.84 (±1.82)	70.52 (±1.81)	69.91 (±1.82)	8.54	<0.001	3.61	0.00	2.63	8.91	3.54	0.00	2.54	8.88
	Diastolic pressure*	80.79 (±2.35)	78.96 (±2.35)	76.37 (±2.35)	3.62	0.03	−1.08	0.28	−4.11	1.19	−2.67	0.01	−6.28	−0.96
	Systolic pressure	122.52 (±2.69)	119.64 (±2.69)	120.14 (±2.7)	0.10	0.91	−0.43	0.67	−3.67	2.36	−0.12	0.90	−3.22	2.84

*Significant fixed effect at *p* < 0.05.

### Auditory steady-state response

**Fig. 1:**
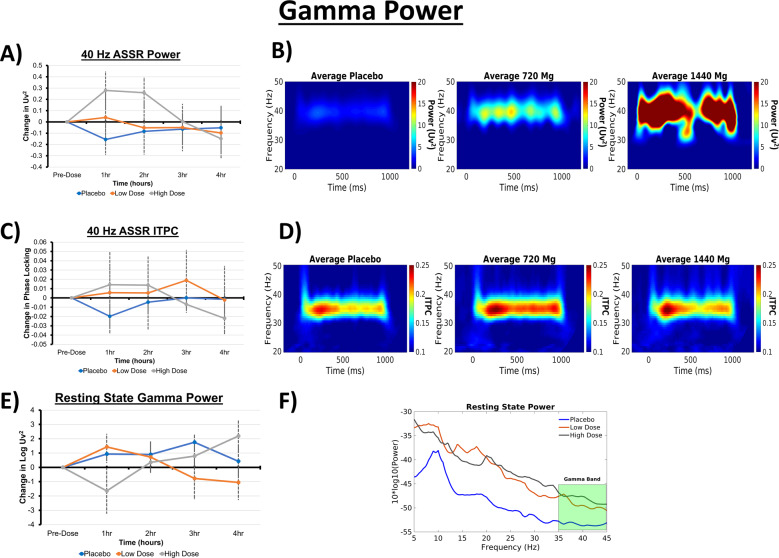
Summary of the measurements of γ-oscillations relative to AV-101 administration. **a** 40 Hz ASSR γ-power at hourly intervals, displayed relative to baseline. **b** Grand average (across time) time-frequency power plot of the response to 40 Hz ASSR presentation for placebo, low, and high doses. **c** 40 Hz ASSR γ-inter-trial phase coherence (ITPC) at hourly intervals, displayed relative to baseline. **d** Grand average (across time) time-frequency ITPC plot of the response to 40 Hz ASSR presentation for placebo, low, and high doses. **e** Change in 10*log10 resting γ-power relative to baseline. **f** Resting-state power spectral density plot averaged across time, for placebo, low, and high doses.

LMM analyses revealed increased 40 Hz ASSR power associated with a significant increase following the high dose, but not the low dose, relative to placebo (see Fig. 1). No significant dose effects were found for 20 or 30 Hz ASSR power. The 40 and 30 Hz inter-trial phase coherence (ITPC) estimates were both increased by the high dose, but not the low dose, relative to placebo.

### Resting-state γ-power

LMM showed that resting-state γ-power was increased by high-dose, but not low-dose, AV-101 relative to placebo (Fig. 1). We anticipated the peak response to occur within 2 h of administration; however, the resting γ-power response to the high dose reached its peak at 4 h. This increases the number of factors that could be driving the significant fixed effect shown in the LMM, which is partially supported by the limits of the 95% confidence interval (lower: −5.98; upper: 0.01). The resting power spectrum was not significantly altered relative to placebo in either dose condition.

### Blood metabolites

**Fig. 2:**
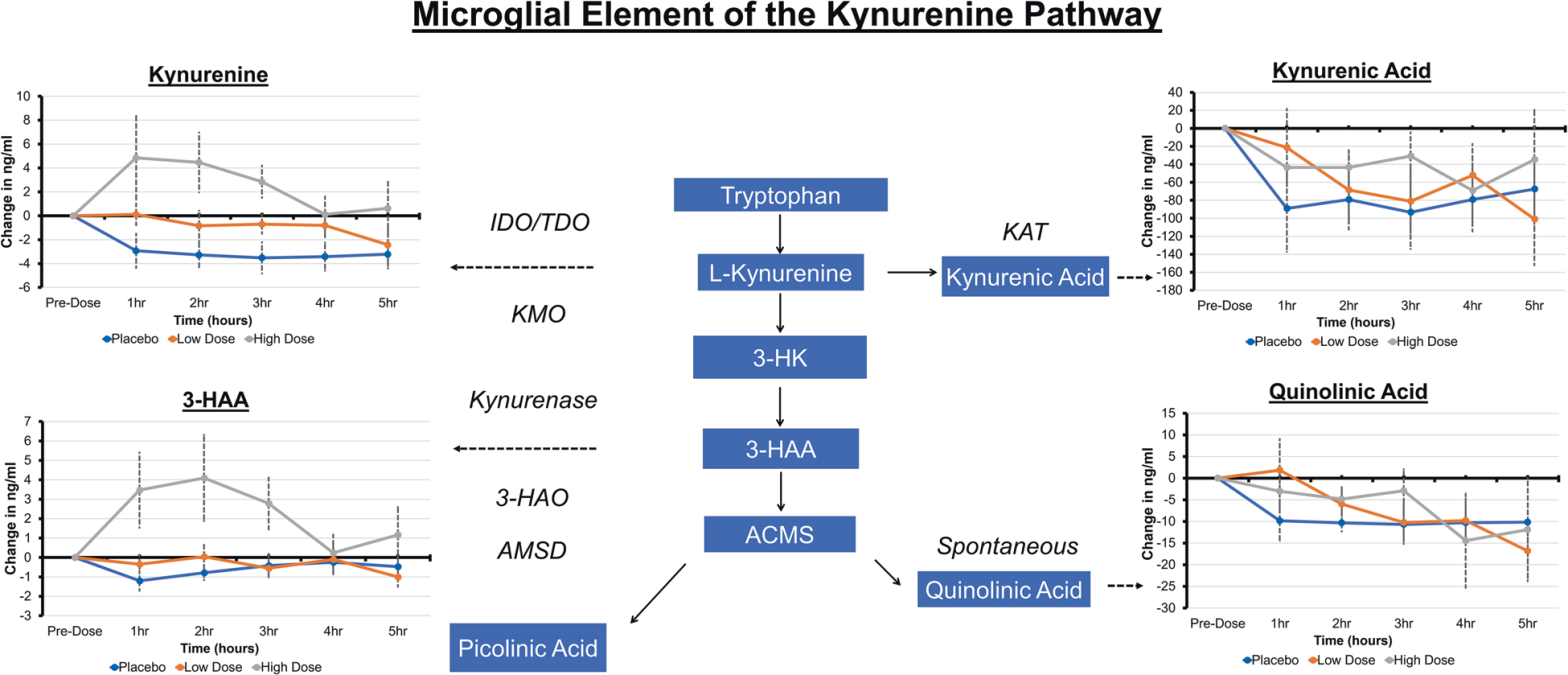
A summary of the blood metabolite findings with respect to their position on the microglial element of the kynurenine pathway. Concentrations are displayed corrected to the baseline timepoint measurement. Variations in the concentration of metabolites during the placebo condition reflect diurnal fluctuations.

**Fig. 3:**
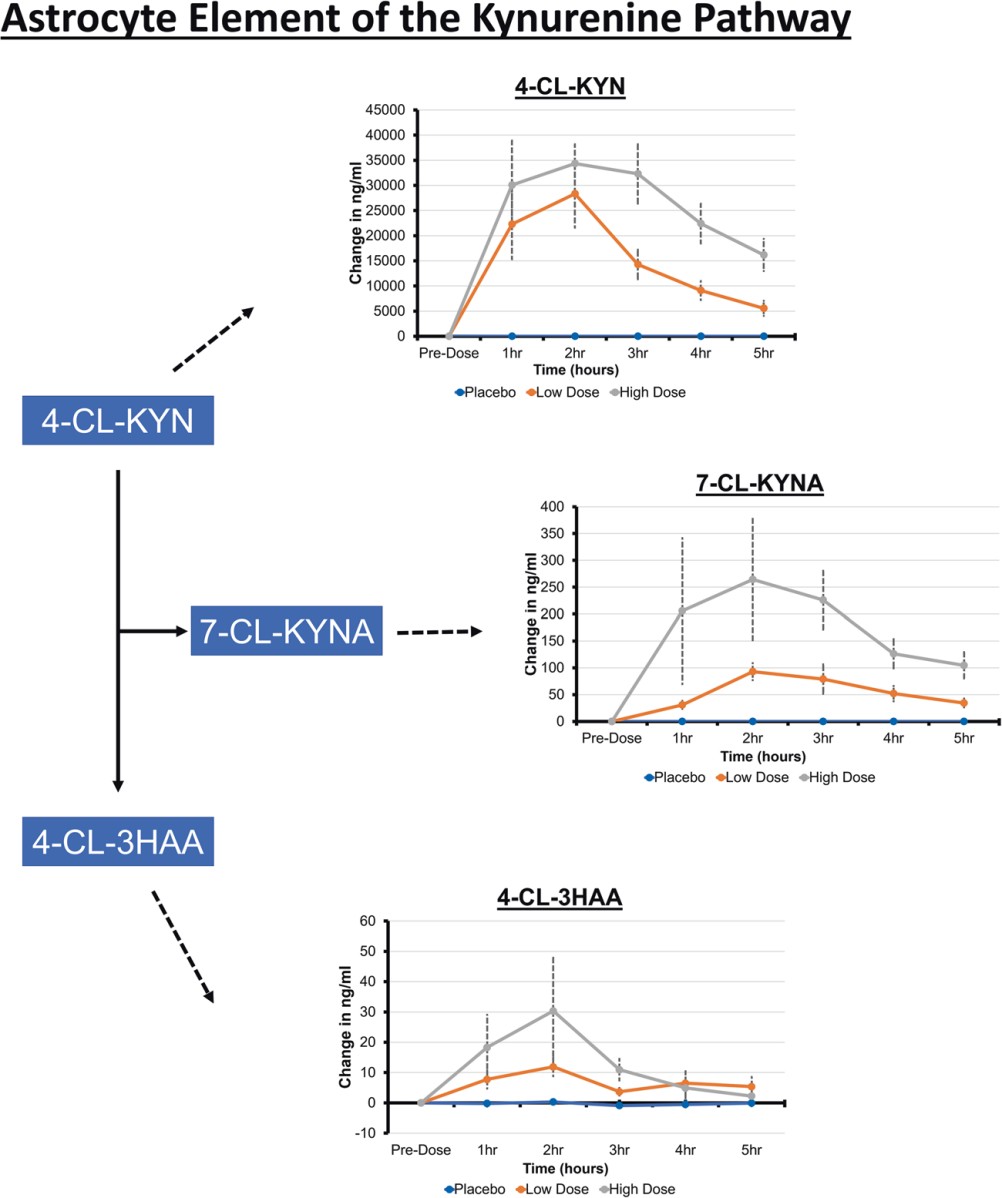
A summary of the blood metabolite findings with respect to their position on kynurenine pathway. Concentrations are displayed corrected to the baseline timepoint measurement.

Blood metabolites are summarized in Figs. 2 and 3. There were dose-related effects on concentrations of 4-Cl-KYN, 7-Cl-KYNA, 4-Cl-3-HAA, KYNA, and 3-HAA. Concentrations of KYN and QUIN did not change significantly. Picolinic acid concentrations were not detectable. In the significant models, the AV-101 high dose was consistently related to greater metabolite concentrations than the placebo and low doses, except for 4-Cl-KYN, which did not demonstrate a significant difference in model fit between high and low doses.

### Adverse events

Participants experienced no serious adverse events. There were no reports of dissociative or psychotic effects. One participant experienced diarrhea with 1440 mg AV-101, which was resolved at the 24 h phone follow-up. One participant experienced “feeling elated” with 720 mg AV-101, which was resolved at the end of the session.

Dose effects on blood pressure and pulse are displayed in Table 1 and Fig. S2 in Supplemental Material. Administration of AV-101 was associated with small reductions in systolic and diastolic blood pressure, and in pulse. LMM analyses revealed that the effects of AV-101 on diastolic blood pressure and pulse, although minimal, were significantly different from placebo. Based on the width of the confidence intervals and significant variation in the random factor (subject intercept), we conclude that these effects may not be generalizable. For the POMS, there was very little variation in scores on any of the items (see Supplemental Fig. S3). We therefore performed analyses on the total POMS score showing no effect of dose or dose by time interaction (for specifics of vital signs and POMS, see Supplemental Material).

## Discussion

The KP is a promising target for the treatment of depression and suicidality, because it inhibits NMDAR activation, with the potential of ketamine-like antidepressant effects without dissociation [8] (see Supplemental Fig. S4). However, AV-101 was not associated with improvement in clinical depression ratings in a recent small TRD study [9] or in a larger adjunct MDD trial (https://www.vistagen.com/news-media/press-releases/detail/130/vistagen-reports-topline-phase-2-results-for-av-101-as-an). In this phase-1 randomized, double-blind, placebo-controlled crossover study, we addressed the question of whether AV-101 at doses of 720 mg (low dose) and 1440 mg (high dose) was sufficient to modulate biochemical and neurophysiological correlates of the KP and NMDARs. Our results indicated that although both the high and low doses were well tolerated, only the high dose (1440 mg) showed clear evidence of target engagement. Effects on neurophysiological markers of NMDAR engagement support the need to study higher doses of AV-101 on NMDAR functioning in a larger clinical sample, if feasible.

The 1440 mg dose of AV-101 was associated with increased power and ITPC of γ-oscillations (Fig. 1), consistent with previous findings on the effects of NMDAR engagement on fast-spiking GABAergic inhibitory interneurons [10, 11, 13, 14, 16]. This probably results from NMDAR-associated suppression of the firing rate of interneurons with simultaneous enhancement of the firing rate of their excitatory pyramidal projections [16]. Optogenetic and molecular biology studies have demonstrated the causal role of NMDA receptor activation on parvalbumin+ neurons in 40 Hz ASSR generation and resting-state γ-oscillations [18, 19]. Pharmacological studies have demonstrated that NMDAR antagonists increase spontaneous γ-band power within 15 min after administration, with a peak at the time of maximum concentration [30]; the increase in γ-power becomes blunted or reversed at around the time of maximum concentration [30]. The return to baseline γ-power depends on the half-life of the drug. ASSR power (40 Hz) has been used in earlier studies to evaluate NMDAR target engagement and to track the rapid pharmacokinetic properties of Ketamine [16, 30]. This provides a robust translatable biomarker, closely reflecting cortical NMDAR receptor function across species [15, 30].

In our study, the increased 40 Hz γ-power could indicate not only brain penetration of AV-101 but also a possible specific effect of 7-Cl-KYNA, a high-affinity NMDAR strychnine-insensitive glycine-binding site competitive antagonist [3–5]. In contrast, the resting-state γ-power results warrant further investigation and could represent one of two potential outcomes. The first of these would be that AV-101 in the 1440 mg dose creates an adaptive shift in GABAergic neural coordination. This could occur on a longer timescale than the effects observed during the ASSR task and might imply that at 1440 mg there is sufficient bioavailability to induce a weak tonic physiological effect that falls below the requirement for a clinical effect. This interpretation is supported by preclinical data, which suggests that AV-101 antidepressant effects in mice were present up to 7 days post administration, at which point 7-CL-KYNA was no longer detectable in the brain [8]. The second interpretation of the data is that our mixed model outcome is driven by a non-cephalic source of noise affecting one or more time points. Irrespective, the significant but otherwise less clear-cut finding for the resting-state data warrants further investigation in a larger sample size and, where possible, should be contrasted with longitudinal recordings in animals following AV-101 dosing. Parallel to the γ-power findings, the 1/*f* distribution of frequency band power in the resting-state spectra (see Supplemental Methods for measurement details) was not significantly altered by AV-101. The 1/*f* power law in EEG is believed to reflect physical and structural aspects of neuronal organization, and has been linked to aggregate spiking of the underlying neuronal population [31, 32]. Changes to the gradient of the slope would indicate a disruption of the excitation/inhibition balance as a result of altered communication between local and distally connected neurons [33]. Thus, the occurrence of increased power in the absence of changes to the 1/*f* properties suggests that AV-101 administration does not result in spill-over across larger neuronal networks. Although the current study focused on markers of short-range communication, we believe that the dose-related changes in γ-power warrant further investigation into the dynamics of long-range communication. Studies of NMDAR antagonism associated with ketamine and phencyclidine have shown divergent effects on α- and θ-dynamics [23–25], suggesting that different mechanisms of receptor binding might impact the wider effects of target engagement.

Our blood metabolite findings (Figs. 2 and 3) demonstrated a dose-dependent response to AV-101 for 4-Cl-3-HAA, as well as KP metabolites 3-HAA and KYNA, without the effects on QUIN and KYN. In post-mortem studies of suicide completers, QUIN concentrations were elevated in the subgenual and supracollosal anterior cingulate cortex [34]. Elevated QUIN concentrations have also been described in the CSF from surviving medically severe suicide attempters after admission to a hospital [35, 36]. In suicide-attempt survivors, QUIN elevation was persistent at follow-up, accompanied by a decrease in concentrations of KYNA in the CSF [36, 37]. NMDAR blockade via AV-101 may alter the balance between QUIN and KYNA. Therefore, NMDAR inhibition-related clinical improvement in treatment- refractory depression patients, such as those in [9], might require KP dysregulation.

AV-101 (4-Cl-KYN, a chlorinated form of KYN) is absorbed by the gut and, in rodents, is transported freely to the brain where it is converted to 7-Cl-KYNA [38] by KAT-II in astrocytes [1, 6, 39, 40] and to 4-Cl-3-HAA by microglia [6]. In preclinical models, 4-Cl-3-HAA dose-dependently inhibited 3-HAO enzyme functioning and ACMS production [6, 38] and lowered brain QUIN [7, 41, 42] even after inflammatory cytokine administration [41, 43]. We found increased plasma 3-HAA with 1440 mg AV-101 without change in plasma QUIN, suggesting that in healthy humans, 4-Cl-3-HAA did not inhibit 3-HAO functioning and ACMS production, at least in the periphery. This may be associated with normal KP regulation in the healthy individuals enrolled in the current study or could reflect differences in KP functioning between humans and animals [6].

Our findings support the clinical pursuit of AV-101 in human subjects. However, potential limitations should be taken into account. We collected data from healthy controls; a population such as suicidal patients is likely to have a complex behavior dysregulation possibly related to altered KP function. Different doses might be required to replicate the engagement of NMDA receptors described in our study, which might also result in different effects on metabolites. In our study design, we chose to control for the effect of time to compensate for the small sample size. This decision was made with the intention that the outcome of the test would reflect a general ability for AV-101 to engage the functional target (ASSR) accounting for time, without a more detailed evaluation of chronological effects of AV-101. The conclusions to our study suggest a conservative outlook for the development of AV-101, where higher doses of AV-101 in healthy controls can modulate NMDA receptor activity (reflected by a well-established biomarker of NMDA antagonism, the ASSR (e.g., see [18]). The difference in effects of 1440 and 720 mg might reflect limitations of AV-101 bioavailability. In human subjects, 1440 mg doses present a plasma C-max level close to the limits reported in previous toxicology studies in rats and dogs [27], preventing study with higher doses. To address this challenge, the combination of AV-101 with probenecid has recently been explored in rodents, demonstrating safe application and up to an 885-fold increase in prefrontal cortex 7-CL-KYNA levels (VistaGen Therapeutics, data on file). In addition, the size and gender balance of our study are also relevant. Although the statistical design of our phase-1 study accounts for random factors, and uses a statistical design that is relatively robust to small sample sizes, a larger and more balanced sample would improve the confidence of the current model fits and address sex-specific effects. Another limitation is the extent to which we can accurately describe the effect of AV-101 on central KP metabolites without CSF data. A single dose of 1,080 mg AV-101 did not increase CSF 7-Cl-KYNA [9], whereas 1440 mg might, based on the effect of 1440 mg AV-101 on γ-band oscillations. The use of CSF markers can identify differences between activation of peripheral and central KPs by AV-101.

In summary, we found that high-dose AV-101 increased NMDAR antagonists 7-Cl-KYNA and KYNA, and increased 4-Cl-3-HAA and 3-HAA without affecting QUIN. AV-101 also increased γ-oscillations consistent with inhibition of NMDAR at GABAergic interneurons after AV-101 brain penetration, potentially due to elevated 7-Cl-KYNA and/or KYNA. The earlier treatment study [9] evaluated functional biomarkers using functional magnetic resonance imaging (fMRI), which did not detect a noticeable change related to NMDA receptor antagonism; however, our findings suggest that ASSR might be sensitive enough to detect functional changes that are missed due to the reduced temporal resolution of fMRI. These findings suggest that AV-101 is a potential intervention for conditions like TRD or suicidality, in which risk is associated with KP dysregulation. This can be addressed by investigating the EEG dose–response relationship and its translation to behavioral and clinical targets, in a larger sample using an appropriate strategy to increase the bioavailability of AV-101. Such changes stand to substantially benefit our understanding of the clinical efficacy of AV-101.

## Funding and disclosure

Funding support and resources and facilities for this study were provided by MEDVAMC Seed Grant (ML). VistaGen Therapeutics provided the AV-101 and placebo capsules, and analyzed AV-101 metabolites. ML has received financial support from the MEDVAMC and the Department of Defense. MAS is an employee of VistaGen Therapeutics. SJM is supported through the use of facilities and resources at the Michael E. Debakey VA Medical Center, Houston, Texas, and receives support from The Menninger Clinic. SJM has served as a consultant to Alkermes, Allergan, Axsome, Clexio Biosciences, Greenwich Biosciences, Intra-Cellular Therapies, Janssen, Perception Neuroscience, Praxis Precision Medicines, Sage Therapeutics, Seelos Therapeutics, and Signant Health. He has received research support from Biohaven Pharmaceuticals and VistaGen Therapeutics. ACS has received grant support from the American Foundation for Suicide Prevention, the VAMC Cooperative Studies Program, the National Institutes of Health, the Department of Defense, and the Linda and John Griffin Family Professorship in Psychiatry. NM, NR, Bylinda Vo-Le, Brittany Vo-Le, and TI declare no potential conflicts of interest.

## Supplementary information

Supplementary Material
