# Supplementary material for: A randomized cross-over trial to define neurophysiological correlates of AV-101 N-methyl-d-aspartate receptor blockade in healthy veterans
Source: Neuropsychopharmacology. 2020 Dec 14;46(4):820–7. doi: 10.1038/s41386-020-00917-z (PMC8027791; doi:10.1038/s41386-020-00917-z)
Supplement: Supplementary file 1 — Supplementary Material [file 41386_2020_917_MOESM1_ESM.docx]

**A Randomized Cross-Over Trial to Define Neurophysiological Correlates of AV-101 N-methyl-D-aspartate Receptor Blockade in Healthy Veterans**

Nicholas Murphy, Nithya Ramakrishnan, Bylinda Vo-Le, Brittany Vo-Le, Mark Smith, Tabish Iqbal, Alan C. Swann, Sanjay J. Mathew, Marijn Lijffijt

**Supplemental Material**

**Methods and Materials**

*Participants*Inclusion Criteria

Participants with eligible to be included in the study if they were between 18 and 64 years old, a US military veteran, and have no history of psychiatric illness. The subject and their partner must both be using at least one medically accepted contraception and randomization, and until one month after a single dose.

Exclusion Criteria

Participants were excluded from the study if they matched any of the following exclusion criteria: A) a history of any axis 1 psychiatric condition; B) a history of psychosis in a first degree family member; C) a history of use of psychoactive medication; D) were engaging in current use of any medication or vitamins at the time of screening (except for the pill – women); E) had a history of use of any substances of abuse (except for alcohol, caffeine, and nicotine); F) tested positive for alcohol and/or illicit substances at initial screening and any study visits; G) a personal history of epilepsy, head injury, stroke, or any primary neurological disorder; H) currently pregnant or nursing; I) diagnoses with any unstable illness; J) demonstration of greater risk for serious adverse events based on abnormal vital signs (determined by the study physician).

*EEG Pre-Processing*

EEG data was processed using in-house Matlab scripts and routines adapted from the EEGLab toolbox [1]. The Matlab code used to process and extract features from the data is available at (<https://github.com/NikMNclUth/AV101-EEG>). Signals were filtered using the combination of a low pass (100 Hz) and high pass (1 Hz) zero-phase FIR filters. Line noise (60 Hz) and its harmonics (120 Hz, 180 Hz, 240 Hz) were attenuated using the Cleanline toolbox [2]. Bad channels were identified using the FASTER bad channel detection routine [3], and replaced after pre-processing using spherical interpolation. To identify and remove the sources of artifacts such as ocular and muscular activity we applied wavelet independent component analysis (wICA), a two-tiered ICA algorithm which filters out high frequency noise at the component level without performing data reduction in the initial instance to improve convergence accuracy on the second application of ICA [4]. ICA components representing blinks, saccades, muscular activity, and additional non-cortical irregularities in the recording were identified and removed using the FASTER and MARA toolboxes [5,6]. Resting state data was split into four-second long segments. Bad trials were identified and removed using the EEGLAB [1] and FASTER [3] routines for bad trial detection. ASSR data was segmented into epochs of duration -1500 ms to 1500 ms, and baseline corrected using the pre-stimulation period mean. Finally, the cleaned data were re-referenced to the common average reference.

*EEG Feature Extraction*

All EEG features were averaged across the fronto-central region of interest (ROI) where ASSR amplitude is typically maximum (*F1, FZ, F2, FC3, FC1, FC2, FC4, C3, C1, CZ, C2, C4,CP1, CP2, CPZ)* [7–9]. ASSR oscillatory power and inter-trial phase coherence (ITPC) were estimated using Morlet wavelets with 10 cycles for the frequencies 1 to 60 Hz. To estimate resting state gamma power, we used the Welch’s power spectral density method (PSD) with a Hanning window (250 samples), and 50% overlap. From this we measured the average power between 35 Hz and 45 Hz from the average power spectrum across the ROI channels. In addition to the resting state power spectrum we estimated the 1/f power law properties for the data using the routine described in [10], which works by identifying peaks in the log-log space power spectrum and then omitting these during the fitting of a two coefficient polynomial. The estimation of the background fit is theorized to represent the level of background activity and is considered an index of the balance of excitatory and inhibitory signaling.

*KP metabolite assays*

The clinical samples were analyzed by Quintara Discovery, Inc. (Hayward, CA). Plasma samples (30 µL) were de-proteinized by adding 100 µL of methanol:acetonitrile (50:50 v/v) with 0.1% formic acid containing internal standards, vortex-mixing for 15 minutes, and centrifugation at 4000 rpm for 15 minutes. The supernatant (65 µL) was diluted with 65 µL of water and analyzed using liquid chromatography with tandem mass spectrometry (LC/MS/MS) with a SCIEX ExionLC ultra performance liquid chromatography (UPLC) coupled with a Qtrap 6500+ mass spectrometer, which was operated in triple-quadruple multiple reaction monitoring mode for positive ionization. The liquid chromatography conditions were as follows: Atlantis T3, 100 x 4.6 mm, 3 µ column (Waters Corporation) at 40° C and a binary solvent system. Mobile phase A was water with 0.5% formic acid and mobile phase B was acetonitrile with 0.1% formic acid, using the flow rate and gradient program as shown in **Table S1**. The calibration curves were prepared by spiking the analytes at a series of concentration levels in the matrix, either water or plasma, which lacked the endogenous compounds or interferences for compound detection and showed suitable compound recovery and matrix effect. The standard curves fitted by linear regression were used to quantify the analytes using Analyst 1.7.0 (SCIEX) software. **Table S2** lists the analytes, their corresponding internal standards, the mass transitions used to detect compounds, and the matrix used to prepare the standard curves.

*Pharmacokinetic analytic methods*

Pharmacokinetic parameters were calculated using Phoenix WinNonLin (v8.1, Certara Corporation). Non-compartmental analysis (NCA) was used to describe concentration-time data from Day 1; concentration data from Days 3-13 were summarized separately. The area under the concentration-time curve (AUC) was determined using the “linear up, log down” trapezoidal method. Concentration values below the limit of quantification were assumed to be 50% of the lower limit of quantification, except for the baseline time point (t=0) when the values below the limit of quantification were set to zero. Lower limit of quantification values for 4-Cl-KYN, 7-Cl-KYNA, and 4-chloro-3-hydroxyanthranilic acid (4-Cl-3-HAA) in plasma were 2.5, 1.25, and 2.5 ng/mL, respectively.

**Table S1**. Liquid chromatography flow rate and gradient program

**Table S2**. Summary of analytes and their corresponding internal standards, mass transitions, and standard curve matrices

|  | | | | | | **Matrix for Standard Curve** | |
| --- | --- | --- | --- | --- | --- | --- | --- |
| **Analyte** | **Q1 (m/z)** | **Q3 (m/z)** | **Internal Standard** | **Q1 (m/z)** | **Q3 (m/z)** | **Plasma Assay** | **CSF Assay** |
| Kynurenine | 209 | 192 | D_6_-Kynurenine | 215 | 198 | Water | Water |
| Picolinic Acid | 124 | 78 | D_6_-Kynurenine | 215 | 198 | Plasma | Plasma |
| 4-Cl-3-HAA | 188 | 170 | D_5_-Kynurenic acid | 195 | 149 | Plasma | Water |
| 7-Cl-KYNA | 224 | 178 | D_5_-Kynurenic acid | 195 | 149 | Plasma | Water |
| 4-Cl-KYN | 243 | 226 | D_5_-Kynurenic acid | 195 | 149 | Plasma | Water |
| 3-HAA | 154 | 136 | D_5_-Kynurenic acid | 195 | 149 | Water | Water |
| Kynurenic Acid | 190 | 144 | D_5_-Kynurenic acid | 195 | 149 | Water | Water |
| Quinolinic Acid | 168 | 78 | D_3_-Quinolinic acid | 171 | 81 | Water | Water |

*Abbreviations*: 4-Cl-KYN: 4-chlorokynurenine; 7-Cl-KYNA: 7-chlorokynurenic acid; 4-Cl-3-HAA: 4-chloro-3-hydroxyanthranilic acid; 3-HAA: 3-hydroxanthranilic acid

**Results**

*CONSORT Flow chart*

N = 18 participants met the eligibility criteria and were recruited to the study. N = 6 participants were withdrawn before the first dose due to screening failures. N = 12 participants were randomized and began the study, N = 2 participants withdrew after completing the first dose (AV101-04 an AV101-07). The completed study consisted of N = 10 participants who received all three doses.


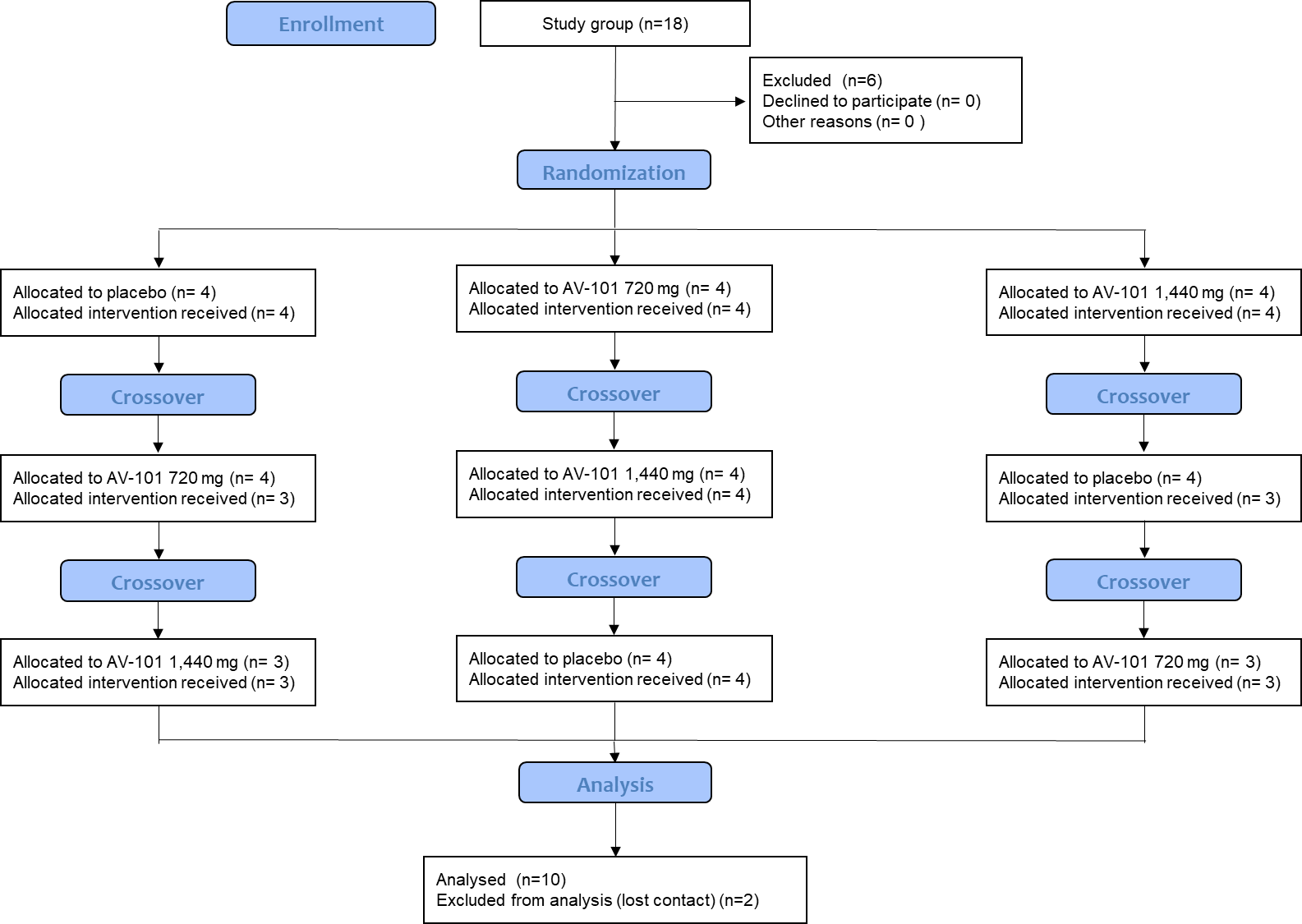


**Figure S1.** Summary of recruitment and randomization

*Vital signs*

**Figure S2**. Summary of the recruitment vital signs data (blood pressure and pulse).

*POMS*


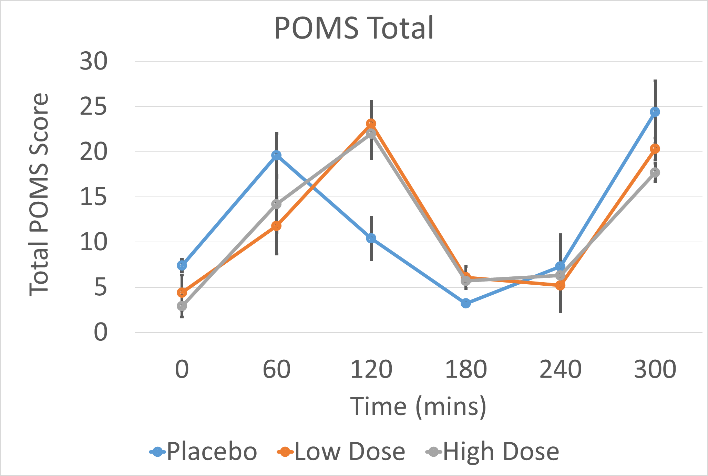

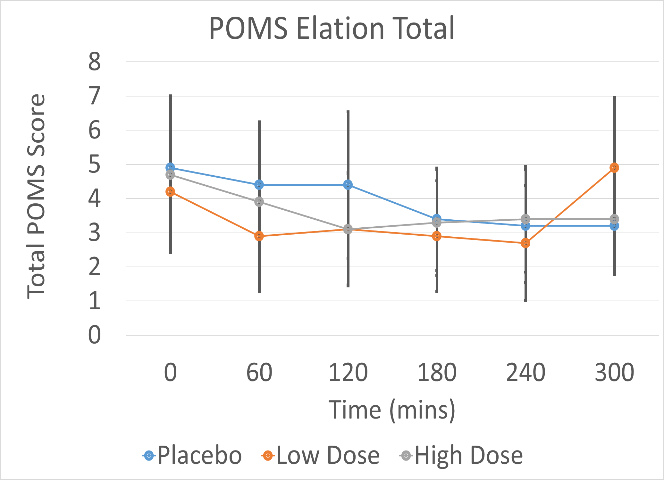


**Figure S3.**  Summary of the POMS data.

**Table S3, Summary of analyte pharmacokinetic properties of 4-CL-KYN and 7-CL-KYNA in the blood plasma following low (720 mg) and high (1440 mg) doses of AV-101. Baseline corrected EEG gamma measurements are included to demonstrate dose related effects at the time of peak concentration in blood plasma.**

|  |  |  |  |  |  |  |
| --- | --- | --- | --- | --- | --- | --- |
| Pharmacokinetic Marker | Units | 720 mg | | 1440 mg | |  |
|  |  | 4-CL-KYN | 7-CL-KYNA | 4-CL-KYN | 7-CL-KYNA |  |
| T-Max | hr | 114 (±34.1) | 120 (±48.9) | 144 (±64.5) | 138 (±40.5) |  |
|  |  |  |  |  |  |  |
| C-Max | ng/mL | 28531.9 (±21462) | 93.05 (±47.6) | 51450 (±21182) | 412.1(±473.3) |  |
|  |  |  |  |  |  |  |
|  |  |  |  |  |  |  |
|  |  |  |  |  |  |  |
|  |  |  |  |  |  |  |

*Pharmacokinetic Analysis*

Assessment of the pharmacokinetic properties T-Max and C-Max for 4-CL-KYN, and 7-CL-KYNA, revealed that the peak plasma concentrations were reached at approximately 2 hours for the 720 mg and 2.5 hours for the 1440 mg dose. The T-Max values are roughly aligned with those presented in [22], which estimated peak plasma concentrations at approximately 1.73 hours. A full assessment of kinetic curve properties is not presented due to insufficient data points.


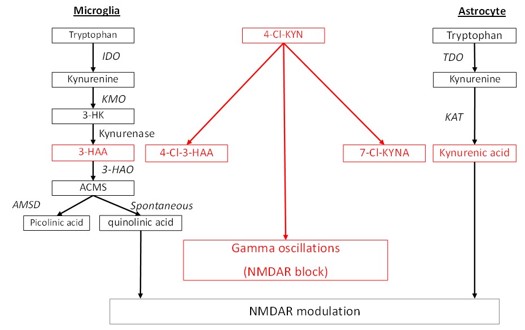


**Figure S4*.*** A schematic overview of the kynurenine pathway. The kynurenine pathway (KP) is a metabolic pathway that catabolizes tryptophan into various physiologically active kynurenines, including NMDAR agonist quinolinic acid (QUIN) and NMDAR antagonist kynurenic acid (KYNA). The KP consists of two enzymatic arms that catabolize tryptophan into kynurenine (KYN) by indoleamine-2,3-dioxygenase (IDO) and by tryptophan-2,3-dioxygenase (TDO). Downstream enzymes catabolize KYN into various physiologically active kynurenines. One pathway relies on kynurenine monooxygenase (KMO) KMO [11] which metabolizes KYN in 3-hydroxykynurenine (3-HK). 3-HK is metabolized to 3-hydroxyanthranilic acid (3-HAA) by kynurenase [12–14] and to 2-amino-3-carboxymuconic-6-semialdehyde (ACMS) by 3-hydroxyanthranilic acid 3,4-dioxygenase (3-HAO) [15]. ACMS is metabolized spontaneously to QUIN, and by amino-beta-carboxymuconate-semialdehyde-decarboxylase (AMSD) to picolinic acid (PIC) [15]. Another pathway relies on kynurenine transferase (KAT) which metabolizes KYN into KYNA. In the periphery, the KMO and KAT pathways exist within the same cell; the KMO and KAT pathways are separated in the brain. In the brain, astrocytes are high in KAT [16] and low in KMO [11], favoring production of KYNA [14,17]; microglia are high in KMO [15]. AV-101 (4-Cl-KYN, a chlorinated form of L-kynurenine) is absorbed by the gut, and, in rodents, transported freely to the brain where it is converted to 7-Cl-KYNA [18] by KAT-II in astrocytes [19–21] and to 4-Cl-3-HAA by microglia [19].

We show that high dose AV-101 increases 7-Cl-KYNA (presumably by astrocytes) and 3-Cl-HAA (presumably by microglia), as well as KYNA and 3-HAA while simultaneously enhancing gamma band oscillations, indicating blockade of NMDAR on interneurons.

**Single Subject 40 Hz ASSR Responses**

The change in gamma power in response to 40 Hz ASSR is presented by time and dose as the percentage change from baseline power.

**Table S4, Individual ASSR gamma power as percentage change from baseline during placebo, 720 mg, and 1440 mg doses of AV-101.**

|  |  |  |  |  |  |
| --- | --- | --- | --- | --- | --- |
| **Subject** | **Dose** | **Percentage Signal Change from Baseline (%)** | | | |
|  |  | **1hr** | **2hr** | **3hr** | **4hr** |
| **5** | **Placebo** | -1.65 | 76.62 | -35.56 | -34.51 |
| **6** | **Placebo** | 81.97 | 27.44 | -19.10 | -83.08 |
| **8** | **Placebo** | 47.33 | 38.18 | -6.73 | 130.68 |
| **9** | **Placebo** | -59.06 | -1.33 | -28.67 | -33.56 |
| **10** | **Placebo** | -16.39 | -35.00 | 144.13 | -23.13 |
| **13** | **Placebo** | -66.90 | -39.75 | 151.21 | 112.78 |
| **14** | **Placebo** | 22.08 | 115.36 | 72.48 | 3.85 |
| **15** | **Placebo** | 61.43 | 28.58 | 2.15 | 1042.85 |
| **17** | **Placebo** | -92.42 | -94.10 | -93.07 | -92.51 |
| **18** | **Placebo** | -38.06 | -25.76 | -12.08 | 22.72 |
| **5** | **Low** | 298.94 | -72.44 | -28.34 | 165.86 |
| **6** | **Low** | 2.42 | -85.60 | -86.65 | -84.30 |
| **8** | **Low** | -88.30 | -16.09 | -32.83 | -33.31 |
| **9** | **Low** | 3050.74 | 3766.55 | 2166.48 | 1562.62 |
| **10** | **Low** | 44.30 | -60.22 | 3.17 | -64.26 |
| **13** | **Low** | 64.40 | 105.88 | 146.45 | 69.23 |
| **14** | **Low** | 9.26 | 95.60 | 64.82 | -25.93 |
| **15** | **Low** | -16.65 | 40.41 | -51.17 | -3.74 |
| **17** | **Low** | -0.21 | 9.58 | -4.45 | -4.32 |
| **18** | **Low** | -40.35 | 8.26 | -4.79 | 18.88 |
| **5** | **High** | 489.89 | 1744.99 | -38.88 | -46.66 |
| **6** | **High** | -20.80 | -14.94 | -17.51 | 64.47 |
| **8** | **High** | 1.09 | -0.31 | -16.39 | -93.00 |
| **9** | **High** | 141.58 | 41.67 | 61.57 | -31.35 |
| **10** | **High** | 2460.32 | -11.67 | 75.60 | 1.62 |
| **13** | **High** | 136.41 | 293.43 | -19.73 | -48.10 |
| **14** | **High** | 47.47 | 28.37 | 43.49 | 24.98 |
| **15** | **High** | -59.20 | 40.03 | -26.53 | -23.94 |
| **17** | **High** | 113.12 | 135.89 | 40.38 | 32.56 |
| **18** | **High** | -30.01 | 17.56 | -24.08 | 16.44 |

**References**

1. Delorme A, Makeig S. EEGLAB: an open source toolbox for analysis of single-trial EEG dynamics including independent component analysis. J Neurosci Methods. 2004;134:9–21.

2. Mullen T. NITRC: CleanLine: Tool/Resource Info. 2012.

3. Nolan H, Whelan R, Reilly RB. FASTER: Fully Automated Statistical Thresholding for EEG artifact Rejection. J Neurosci Methods. 2010;192:152–162.

4. Castellanos, N.P., & Makarov, V.A. (2006). "Recovering EEG brain signals: Artifact suppression with wavelet enhanced independent component analysis" J. Neurosci. Methods 158, 300-312

5. Winkler I, Haufe S, Tangermann M. Automatic classification of artifactual ICA-components for artifact removal in EEG signals. Behav Brain Funct. 2011;7:30.

6. Winkler I, Brandl S, Horn F, Waldburger E, Allefeld C, Tangermann M. Robust artifactual independent component classification for BCI practitioners. J Neural Eng. 2014;11:035013.

7. Spencer KM, Niznikiewicz MA, Nestor PG, Shenton ME, McCarley RW. Left auditory cortex gamma synchronization and auditory hallucination symptoms in schizophrenia. BMC Neurosci. 2009;10:85.

8. Schwarz DWF, Taylor P. Human auditory steady state responses to binaural and monaural beats. Clin Neurophysiol. 2005;116:658–668.

9. Saupe K, Schröger E, Andersen SK, Müller MM. Neural mechanisms of intermodal sustained selective attention with concurrently presented auditory and visual stimuli. Front Hum Neurosci. 2009;3:58.

10. Colombo MA, Napolitani M, Boly M, Gosseries O, Casarotto S, Rosanova M, et al. The spectral exponent of the resting EEG indexes the presence of consciousness during unresponsiveness induced by propofol, xenon, and ketamine. NeuroImage. 2019;189:631–644.

11. Bohár Z, Toldi J, Fülöp F, Vécsei L. Changing the face of kynurenines and neurotoxicity: therapeutic considerations. Int J Mol Sci. 2015;16: 9772–9793. doi:10.3390/ijms16059772

12. Adams S, Braidy N, Bessede A, Bessesde A, Brew BJ, Grant R, et al. The kynurenine pathway in brain tumor pathogenesis. Cancer Res. 2012;72: 5649–5657. doi:10.1158/0008-5472.CAN-12-0549

13. Campbell BM, Charych E, Lee AW, Möller T. Kynurenines in CNS disease: regulation by inflammatory cytokines. Front Neurosci. 2014;8. doi:10.3389/fnins.2014.00012

14. Guillemin GJ. Quinolinic acid, the inescapable neurotoxin. FEBS J. 2012;279: 1356–1365. doi:10.1111/j.1742-4658.2012.08485.x

15. Schwarcz R, Bruno JP, Muchowski PJ, Wu H-Q. Kynurenines in the mammalian brain: when physiology meets pathology. Nat Rev Neurosci. 2012;13: 465–477. doi:10.1038/nrn3257

16. Rzeski W, Kocki T, Dybel A, Wejksza K, Zdzisińska B, Kandefer-Szerszeń M, et al. Demonstration of kynurenine aminotransferases I and II and characterization of kynurenic acid synthesis in cultured cerebral cortical neurons. J Neurosci Res. 2005;80: 677–682. doi:10.1002/jnr.20505

17. Oxenkrug G. Serotonin-kynurenine hypothesis of depression: historical overview and recent developments. Curr Drug Targets. 2013;14: 514–521.

18. Hokari M, Wu HQ, Schwarcz R, Smith QR. Facilitated brain uptake of 4-chlorokynurenine and conversion to 7-chlorokynurenic acid. Neuroreport. 1996;8: 15–18.

19. Guidetti P, Wu HQ, Schwarcz R. In situ produced 7-chlorokynurenate provides protection against quinolinate- and malonate-induced neurotoxicity in the rat striatum. Exp Neurol. 2000;163: 123–130. doi:10.1006/exnr.1999.7284

20. Wu HQ, Salituro FG, Schwarcz R. Enzyme-catalyzed production of the neuroprotective NMDA receptor antagonist 7-chlorokynurenic acid in the rat brain in vivo. Eur J Pharmacol. 1997;319: 13–20.

21. Kiss C, Ceresoli-Borroni G, Guidetti P, Zielke CL, Zielke HR, Schwarcz R. Kynurenate production by cultured human astrocytes. J Neural Transm (Vienna). 2003;110: 1–14. doi:10.1007/s00702-002-0770-z

22. Wallace M, White A, Grako KA, Lane R, Cato AJ, Snodgrass HR. Randomized, double-blind, placebo-controlled, dose-escalation study: Investigation of the safety, pharmacokinetics, and antihyperalgesic activity of l-4-chlorokynurenine in healthy volunteers. Scand J Pain. 2017;17:243–251.
